# Task‐shifting from healthcare staff to patients is necessary for the future

**DOI:** 10.1111/aogs.14836

**Published:** 2024-04-16

**Authors:** Sebastian Gidlöf

**Affiliations:** ^1^ Department of Gynecology and Reproductive Medicine Karolinska University Hospital Stockholm Sweden; ^2^ Department of Women's and Children's Health Karolinska Institutet Stockholm Sweden; ^3^ Department of Clinical Science, Intervention and Technology – CLINTEC Karolinska Institutet Stockholm Sweden

The healthcare system is facing unprecedented challenges in the near future with a rapid increase in the shortage of staff. This shortage is not only explained by an aging population. Advances in medical technology and treatment has increased the demand for health services as more conditions can be treated and new more complicated therapies are available. As the field of medicine develops, the need for highly specialized physicians, surgeons, nurses and allied health professionals increases. Difficulties in retaining experienced staff is an emerging problem and employers already struggle to uphold good work conditions. This may be even harder when the shortage of staff continues to increase. Taking these and several other factors into consideration it becomes clear that new ways to manage healthcare are needed.

There are several good initiatives to direct resources, and making evidence‐based and patient‐centered good clinical choices has become ever more important. However, the shortage in staff is destined to drive new developments where task‐shifting from healthcare workers to patients and their relatives will be necessary.

In gynecology, self‐sampling of human papillomavirus (HPV) testing has already been implemented in several nations.^1^ Although it was introduced to increase participation rate, in fact, it may also reduce the workload. This is a good example of the traditional task‐shift development from healthcare staff to patients themselves. Also, self‐testing for certain sexually transmitted diseases (STD) has been implemented.^2^ Both these examples display how tasks can be moved from a strained healthcare workforce to patients, thereby making it possible to direct resources into other areas.

In obstetrics, methods where fetal scanning can be interpreted by artificial intelligence (AI) are being developed.^3^ This will save thousands of working hours for obstetricians, midwives and sonographers thus enabling the workforce to be directed into other more specialized tasks. Similarly, fertility treatment monitoring is another field where standardized ultrasound examinations, performed by the patients themselves, could be used to aid decision‐making.

Furthermore, as equipment for self‐monitoring becomes more instructive and easier to use, what we today consider to be absolute indications for in‐patient care is likely to move to ambulatory care. One example in antenatal medicine is the use of cardiotocography (CTG) at home.^4^ This opens for ambulatory care; for example, in patients with preterm rupture of membranes that have historically been hospitalized for weeks.

The industry of so‐called Fem‐tech applications is growing fast,^5^ with more and more software being developed to aid patients in monitoring treatments, menstrual cycle, and fertility. Ultimately, this may reduce the need for doctor's appointments and prolong the time between formal visits. If used in the right way these platforms may therefore move tasks from healthcare professionals to patients.

In addition to changing the mindset of healthcare workers and managers there are several other challenges which need to be overcome. It is necessary that all technical equipment used by patients are evaluated and approved for use by not formally trained professionals. This needs to be addressed by both manufacturers and regulatory bodies without further delay. There are also several ethical considerations. For example, all patients are not equally equipped with the necessary background and abilities to pick up training on how to handle medical tasks. Safety is another concern, and it is important to ascertain that the risk for misuse and misinterpretation is reduced to not cause harm.

Not only is task‐shifting from healthcare staff to patients necessary to reduce the shortage in staff, but it also has the potential to improve the quality of healthcare. It has, for instance, been shown that the uptake for cervical cancer screening increased when implementing self‐sampling^1^ and that the sensitivity for chlamydia sampling is better when patients take the tests themselves.^2^ We should therefore try to aid the development of task‐shifting to patients. It is also necessary to secure accessible and high‐quality healthcare in the future.
